# Validation of Two Prognostic Gene Scores in Patients Undergoing Liver Resection for Hepatocellular Carcinoma

**DOI:** 10.1016/j.jceh.2025.102544

**Published:** 2025-03-11

**Authors:** Stinna D. Schnabl, Jeanett Klubien, Colm J. O'Rourke, Sophie Bull Nordkild, Jan-Michael Kugler, Susanne Dam Nielsen, Jesper B. Andersen, Hans-Christian Pommergaard

**Affiliations:** ∗ Department of Surgery and Transplantation, Copenhagen University Hospital, Rigshospitalet, Denmark; † Hepatic Malignancy Surgical Research Unit (HEPSURU), Department of Surgery and Transplantation, Rigshospitalet, Copenhagen University Hospital, Denmark; ‡ Biotech Research and Innovation Centre (BRIC), Department of Health and Medical Sciences, University of Copenhagen, Copenhagen, Denmark; § Institute for Molecular and Cellular Medicine, University of Copenhagen, Panum Institute, Copenhagen, Denmark; ‖ Viro-immunology Research Unit, Department of Infectious Diseases, Copenhagen University Hospital, Rigshospitalet, Denmark; ¶ Institute for Clinical Medicine, University of Copenhagen, Panum Institute, Copenhagen, Denmark

**Keywords:** biomarker, gene signature, liver cancer, liver resection, prognosis

## Abstract

**Background/Aims:**

Several prognostic gene signatures have been proposed as predictors of the prognosis of hepatocellular carcinoma (HCC), yet none are implemented in the clinical setting. We aimed to validate two gene scores previously derived from European cohorts.

**Methods:**

The patients who underwent liver resection for HCC at Copenhagen University Hospital, Rigshospitalet from 2014 to 2018 were included. RNA sequencing determined the expression of genes in the ‘*5-gene score*’ (*HN1, RAN, RAMP3, KRT19, TAF9B*) and ‘*HepatoPredict’* (*CLU, DPT, SPRY2, CAPSN1*). Univariable Cox regression assessed associations with overall and disease-free survival. These parameters were also analyzed in the The Cancer Genome Atlas Liver Hepatocellular Carcinoma (TCGA-LIHC) (n = 359) and National Institute of Health (NIH) (n = 178) cohorts.

**Results:**

Among 51 patients (88% male), 59% had no underlying liver disease and 25% had cirrhosis. No individual genes were significantly associated with overall survival in the Danish cohort. In the TCGA-LIHC cohort, *CLU* was linked to better overall survival, and in the NIH cohort, high expression of *SPRY2* was associated with poorer overall survival. In the TCGA-LIHC cohort, *HN1*, *RAN*, and *TAF9B* were associated with poorer overall survival, while *RAMP3* was linked to better overall survival. No genes were associated with disease-free survival.

**Conclusion:**

Few individual genes significantly predicted survival in the larger cohorts, and none in the Danish cohort. However, the clinical implication of this needs further investigation.

## Graphical abstract

**Figure:**
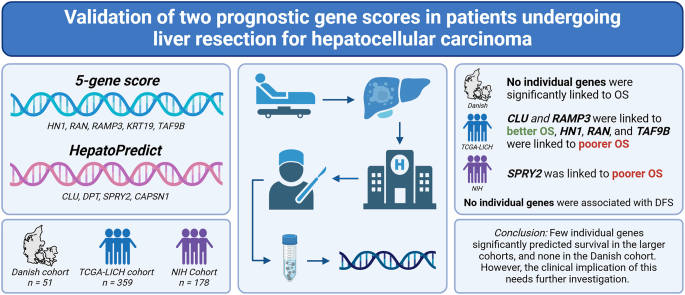

Primary liver cancer is the sixth most common cancer type worldwide and the third most prevalent cause of cancer-related death. Hepatocellular carcinoma (HCC) accounts for approximately 80% of these cases.^1^ The strongest risk factor for HCC is cirrhosis derived from any major recognized etiology. Major causes of cirrhosis, and hence HCC, include chronic infection with the hepatitis B virus (HBV) or hepatitis C virus (HCV) as well as excess alcohol consumption, diabetes mellitus type 2 (DMT2), and metabolic dysfunction-associated steatotic liver disease (MASLD).^2^, ^3^, ^4^, ^5^ However, the etiology of HCC differs drastically depending on the region.^1^^,^^3^ Incidence and mortality of HCC are rising in the United States and Europe in part due to the increasing occurrence of obesity and DMT2 as well as substantial alcohol consumption causing liver dysfunction.^6^^,^^7^

Potentially curative treatment options, including liver resection, ablation, and liver transplantation, may be offered to patients at an early disease stage. However, many patients are diagnosed too late to be eligible for surgery, since symptoms often arise late, and current surveillance strategies can be flawed.^3^^,^^8^ The gold standard for patients with preserved liver function and a solitary lesion larger than 3 cm is liver resection,^8^^,^^9^ but only 5–10% of all HCC patients are eligible candidates for this procedure.^3^ Furthermore, the recurrence rate after liver resection is reported up to 70% within five years.^2^^,^^3^

Numerous biomarkers have previously shown a correlation with the prognosis of HCC.^3^^,^^4^ Scores combining clinical variables and biomarkers for patients at risk of developing HCC, such as GALAD, have shown promise in surveillance of HCC development.^3^^,^^10^ Other scores including biomarkers may help predict the prognosis of HCC as well as allow for a personalized treatment strategy by identifying patients who may benefit from surgery, e.g., low risk of recurrence. However, despite the vast research in prognostic biomarkers, most results are not validated, and only α-fetoprotein is implemented for HCC surveillance in a clinical setting, emphasizing the need for further research in this field.^4^

The literature on gene signatures for HCC is evolving, and while there is no validated and established gene score, we focused on two scores from France and Portugal, respectively, with similar characteristics to our population. Nault *et al.* presented a *5-gene score* able to predict overall and disease-free survival of patients treated with liver resection for HCC.^11^ The 5-gene score was based on the collective expression of *hematological and neurological expressed 1* (*HN1), ras-related nuclear protein (RAN), receptor activity modifying protein 3 (RAMP3), keratin 19 (KRT19)*, and *TATA-box binding protein associated factor 9* (*TAF9B)*. Pinto-Marques *et al.* on the other hand presented an algorithm for predicting the prognosis of patients after liver transplantation for HCC, called HepatoPredict. It is based on four genes: *clusterin* (*CLU), dermatopontin (DPT), sprouty RTK signaling antagonist 2 (SPRY2),* and *calpain small subunit 1 (CAPSN1).*^12^

The genetic landscape of HCC is highly dependent on the underlying liver disease that leads to HCC, and this varies depending on the geographical region.^13^ Thus, the identified gene scores may not apply in other populations, where the prevalence of cirrhosis and chronic hepatitis B and C infections is low. Therefore, this study aims to validate these two gene scores in different cohorts of patients treated with liver resection for HCC. We hypothesized that the HepatoPredict and *5-gene scores* could predict the prognosis of patients undergoing liver resection for HCC in all cohorts.

## METHODS

The study was approved by the regional ethics committee (journal-nr.: H-18015944, H-17027451) and the Danish Data Protection Agency (RH-2018-29, I-Suite nr.: 6184).

This was a retrospective, observational, single-center cohort study including patients treated with liver resection for HCC at Rigshospitalet, Copenhagen University Hospital, Denmark, with stored freshly frozen tumor tissue available in The Danish Cancer Biobank. The study was approved by the regional ethics committee (journal-nr.: H-18015944, H-17027451) and the Danish Data Protection Agency (RH-2018-29, I-Suite nr.: 6184). The reporting follows STrengthening the REporting of Genetic Association studies (STREGA) guidelines.^14^

Patients included in the study were subjected to resection between May 2014 and January 2018; the end of the follow-up was March 22, 2024.

The treatment decision was based on Barcelona Clinic Liver Cancer (BCLC) staging^8^ and discussion at a multidisciplinary team conference. The patients were offered standardized follow-up with abdominal CT scans after 3, 6, 9, 12, 24, 36, 48, and 60 months.

Tumor tissues from the Danish Cancer Biobank were examined for gene expression levels. RNA from tumor tissue was isolated with Trizol, following the manufacturer’s guidelines. RNA sequencing services were outsourced to BGI Genomics (Copenhagen, Denmark), and sample analysis was conducted using the BGISEQ-500 platform.

Patient and tumor characteristics were obtained from electronic medical records including the pathology registry (Patobank). These variables include patient characteristics (age, sex, etiology of liver disease, and BCLC stage), and pathology (tumor size, number of tumors, tumor stage, microvascular invasion, free resection margins, and METAVIR score). The endpoints of the study were overall and disease-free survival.

Gene expressions were also tested in The Cancer Genome Atlas Liver Hepatocellular Carcinoma (TCGA-LIHC)^15^ and the US National Institute of Health (NIH)^16^ cohorts, comprising 359 and 178 patients, respectively, all of whom underwent liver resection for HCC. The TCGA-LIHC and NIH cohorts were published in 2017 and 2010, respectively, and both of these publications reported multi-omics data generated using resected tissues from these cohorts. Transcriptome data and limited anonymized clinical data have been released to the clinical and scientific communities.^17^^,^^18^

For the TCGA-LIHC cohort, we downloaded the processed RNA-seq data from GDAC Firehose (Broad). This cohort comprised resected HCC specimens from patients residing in the United States. Clinical data were downloaded from cBioPortal. Overall survival information was reported for 359 patients with transcriptome data. Disease-free survival information was reported for 272 patients with transcriptome data. Gene expression was quantified in RNA-Seq by Expectation-Maximization (RSEM)–normalized counts.

For the publicly available NIH cohort, raw gene expression array data (Affymetrix Human Genome U133A 2.0 Array) were downloaded from Gene Expression Omnibus (GSE14520). This cohort comprised resected HCC specimens from patients predominantly from China. Information on disease-free and overall survival was available for 178 patients. Data were subjected to robust multiple-array average (RMA) normalization (‘*oligo*’ R package) and gene expression was quantified by log-scaled signal intensity in arbitrary units.

### Statistical Analyses

The study size was based on the available tumor tissue; therefore, no power calculation was done prior to inclusion. Continuous variables were reported with median and range or interquartile range and categorical variables with frequencies and percentages. The association between gene expression (treated as a continuous variable) and outcome (disease-free or overall survival) was evaluated by univariable Cox proportional hazards regression modeling (calculated using the ‘*survival’* R package). No violation of the proportional hazard was found for any of the variables. R, 4.2.2 was used for statistical analyses. Results are reported by hazard ratio (HR) and 95% confidence interval (CI). A *P* value below 0.05 was considered statistically significant. Results were visualized by forest plots using the ‘*forestplot*’ R package.

## RESULTS

### Patient Characteristics

In total, 117 patients were resected for HCC in the inclusion period. Of these, 54 patients had freshly frozen tumor tissue stored in the Danish Cancer Biobank. The tissue was primarily collected during the later part of the period when the procedure was implemented as a standard of care. One patient was excluded due to receiving liver transplantation. One patient died 41 days postprocedural due to liver failure related to the surgery. Transcriptomic analyses aiming at oncological biomarkers were thus omitted due to the lack of information on oncological outcomes. This left 51 patients to be included in this study. The median follow-up was 56 months (range 2–118).

Baseline characteristics of the cohort from Rigshospitalet are shown in Table 1. Of the total patients included, 45 (88%) were male. Thirty patients (59%) had no known underlying liver disease at the time of surgery, and 10 patients (20%) had hepatitis C virus. A total of eight patients (16%) had suspected alcoholic cirrhosis preoperatively; however, only three (6%) had confirmed histological cirrhosis; the remaining patients had fibrosis. Moreover, 13 patients (25%) had cirrhosis and 28 patients (55%) had fibrosis according to the METAVIR score.

**Table 1:** Baseline Characteristics at Time of Liver Resection for Hepatocellular Carcinoma.

	n = 51
Age in years, median (IQR)	69 (63–74)
Sex, n (%)	
Male	45 (88)
Female	6 (12)
Etiology of liver disease, n (%)	
No known underlying liver disease	30 (59)
Hepatitis C virus	10 (20)
Alcoholic liver disease^a^	8 (16)
Hemochromatosis	2 (4)
Nonalcoholic steatohepatitis	1 (2)
Liver function^b^, n (%)	
Normal	10 (20)
Fibrosis	28 (55)
Cirrhosis	13 (25)
Barcelona clinic liver cancer prognosis and treatment strategy stage, n (%)	
0	3 (6)
A	40 (78)
B	8 (16)
Number of tumors, n (%)	
1	43 (84)
2	6 (12)
3	2 (4)
Diameter of largest tumor, mm, median (range)	45 (17–250)
Stage, n (%)	
pT1	24 (47)
pT1b	4 (8)
pT2	9 (18)
pT2a	1 (2)
pT3	1 (2)
pT3a	8 (16)
pT3b	3 (6)
pT4	1 (2)
Microvascular invasion, n (%)	17 (33)
Free resection margins, n (%)	41 (80)

a Patients had suspected alcoholic cirrhosis preoperatively; however, only 3 (6) had confirmed histological cirrhosis; the remaining patients had fibrosis.

b Based on METAVIR scores.

Within the study period, 34 (67%) developed recurrence and 7 patients (14%) died without any noted recurrence. For one patient, residing outside of Denmark, information on recurrence is lacking.

In the TCGA-LIHC cohort, the median age was 61 (range 16–90) and 239 patients (67%) were male. Additionally, 247 patients (69%) had AJCC stage I-II disease, 88 (25%) had stage III-IV disease, and 21 patients (6%) did not have available data regarding tumor stage. In the NIH cohort, the median age was 50 (range 21–74), and 152 patients (85%) were male. Moreover, 139 patients (78%) had AJCC stage I-II disease, 37 (21%) had stage III-IV disease, and two patients (1%) did not have available data regarding tumor stage. Clear information about the etiology or cirrhosis was not available for these cohorts.

### Association Between Gene Expression and Prognosis

**Figure 1:**
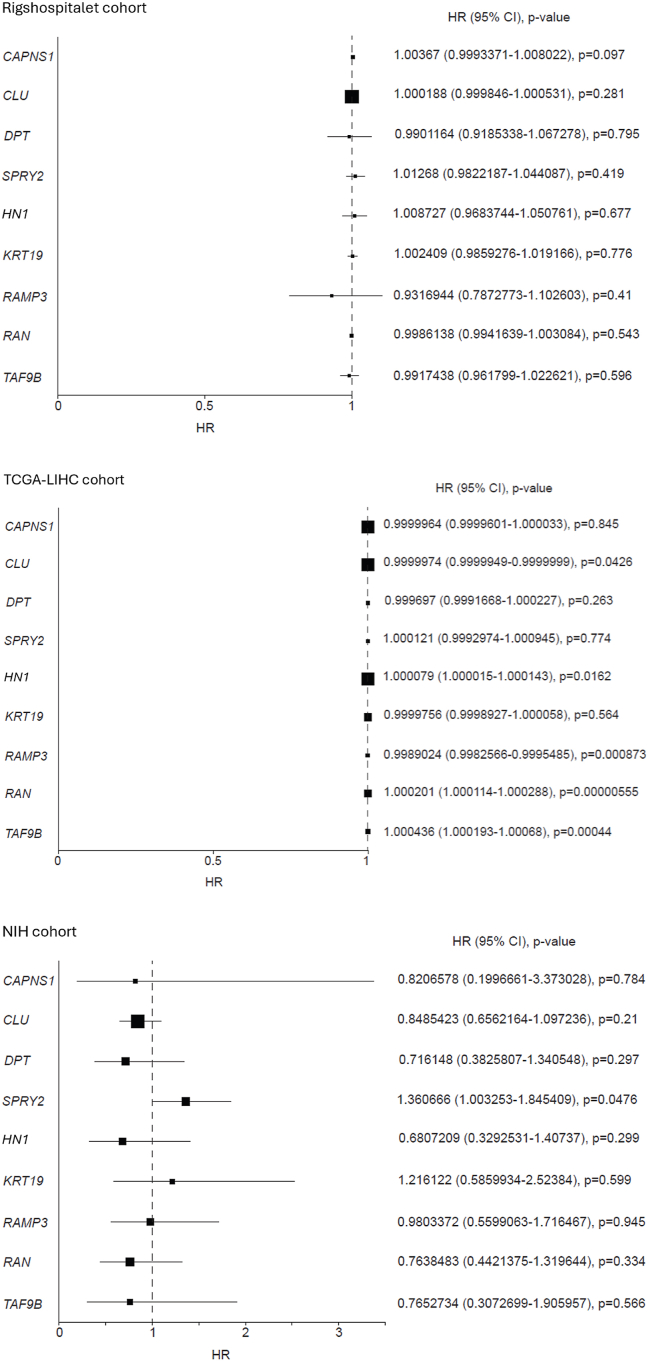
Overall survival. Association of the investigated genes to overall survival in patients with hepatocellular carcinoma treated with liver resection in three cohorts. The plot displays estimates of the hazard ratio (HR) for each gene along with the corresponding 95% confidence intervals (CI) and *P* value.

Results on overall survival are summarized in Table 2 and illustrated in Figure 1. Regarding both gene scores, high expression of none of the included genes were individually significantly associated with overall survival in the Danish cohort. Of the four genes from the HepatoPredict score, high expression of *CLU* was associated with better overall survival in the TCGA-LIHC cohort (HR 1.00000, 95% CI: 0.99999–1.00000, *P* = 0.043), and high *SPRY2* expression was associated with poor overall survival in the NIH cohort (HR 1.36067, 95% CI: 1.00325–1.84541, *P* = 0.048).

**Table 2:** Univariable Cox Regression Analyses of Overall Survival for Patients Treated With Liver Resection.

OS	Rigshospitalet cohort			TCGA-LIHC cohort			NIH cohort		
Variable	HR	95% CI	*P* value	HR	95% CI	*P* value	HR	95% CI	*P* value
*CAPNS1*	1.00367	0.9993–1.00802	0.097	1.00000	0.99996–1.00003	0.845	0.82066	0.19967–3.37303	0.784
*CLU*	1.00019	0.99985–1.00053	0.281	1.00000	0.99999–1.00000	0.043∗	0.84854	0.65622–1.09724	0.210
*DPT*	0.99012	0.91853–1.06728	0.795	0.99970	0.99917–1.00023	0.263	0.71615	0.38258–1.34055	0.297
*SPRY2*	1.01268	0.98222–1.04409	0.419	1.00012	0.99930–1.00095	0.774	1.36067	1.00325–1.84541	0.048∗
*HN1*	1.00873	0.96837–1.05076	0.677	1.00008	1.00002–1.00014	0.016∗	0.68072	0.32925–1.40737	0.299
*KRT19*	1.00241	0.98593–1.01917	0.776	0.99998	0.99989–1.00006	0.564	1.21612	0.58599–2.52384	0.599
*RAMP3*	0.93169	0.78728–1.10260	0.410	0.99890	0.99826–0.99955	0.001∗	0.98034	0.55991–1.71647	0.945
*RAN*	0.99861	0.99416–1.00308	0.543	1.00020	1.00011–1.00029	<0.001∗	0.76385	0.44214–1.31964	0.334
*TAF9B*	0.99174	0.96180–1.02262	0.596	1.00044	1.00019–1.00068	<0.001∗	0.76527	0.30727–1.90596	0.566

OS, overall survival; HR, hazard ratios; 95% CI, 95% confidence interval; NIH, National Institute of Health; TCGA-LIHC, The Cancer Genome Atlas Liver Hepatocellular Carcinoma. The top four genes belong to the HepatoPredict score and the bottom five belong to the 5-gene score. All genes were tested in the Danish cohort (Rigshospitalet), the TCGA-LIHC cohort, and the NIH cohort.

∗*P* values of 0.05 or less were considered statistically significant.

High expression of four of the five investigated genes from the 5-gene score were found to be significantly associated with overall survival in The Cancer Genome Atlas Liver Hepatocellular Carcinoma (TCGA-LIHC) cohort: *HN1* (HR 1.00008, 95% CI: 1.00002–1.00014, *P* = 0.016), *RAMP3* (HR 0.99890, 95% CI: 0.99826–0.99955, *P* = 0.001), *RAN* (HR 1.00020, 95% CI: 1.00011–1.00029, *P* < 0.001), and *TAF9B* (HR 1.00044, 95% CI: 1.00019–1.00068, *P* < 0.001). However, none were significant in the NIH cohort.

**Figure 2:**
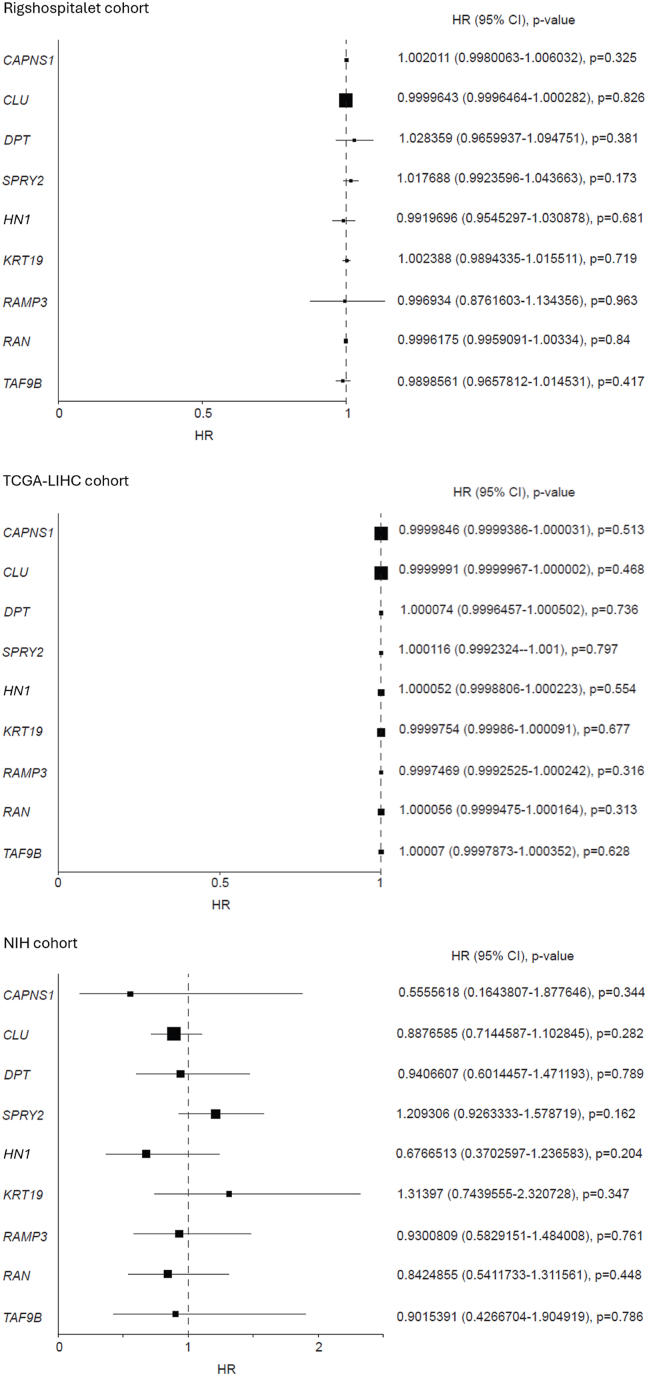
Disease-free survival. Association of the investigated genes to disease-free survival in patients with hepatocellular carcinoma treated liver with resection in three cohorts. The plot displays estimates of the hazard ratio (HR) for each gene along with the corresponding 95% confidence intervals (CIs) and *P* value.

None of the investigated genes were found to be significantly associated with disease-free survival in any of the three cohorts (Table 3, Figure 2).

**Table 3:** Univariable Cox Regression Analyses of Disease-free Survival for Patients Treated With Liver Resection.

DFS	Rigshospitalet cohort			TCGA-LIHC cohort			NIH cohort		
Variable	HR	95% CI	*P* value	HR	95% CI	*P* value	HR	95% CI	*P* value
*CAPNS1*	1.00201	0.99801–1.00603	0.325	0.99998	0.99994–1.00003	0.513	0.55556	0.16438–1.87765	0.344
*CLU*	0.99996	0.99965–1.00028	0.826	1.00000	1.00000–1.00000	0.468	0.88766	0.71446–1.10285	0.282
*DPT*	1.02836	0.96599–1.09475	0.381	1.00007	0.99965–1.00050	0.736	0.94066	0.60145–1.47119	0.789
*SPRY2*	1.01769	0.99236–1.04366	0.173	1.00012	0.99923–1.00200	0.797	1.20931	0.92633–1.57872	0.162
*HN1*	0.99197	0.95453–1.03088	0.681	1.00005	0.99988–1.00022	0.554	0.67665	0.37026–1.23658	0.204
*KRT19*	1.00239	0.98943–1.01551	0.719	0.99998	0.99986–1.00009	0.677	1.31397	0.74396–2.32073	0.347
*RAMP3*	0.99693	0.87616–1.13436	0.963	0.99975	0.99925–1.00024	0.316	0.93008	0.58292–1.48401	0.761
*RAN*	0.99962	0.99591–1.00334	0.840	1.00006	0.99995–1.00017	0.313	0.84249	0.54117–1.31156	0.448
*TAF9B*	0.98986	0.96578–1.01453	0.417	1.00007	0.99979–1.00035	0.628	0.90154	0.42667–1.90492	0.786

DFS, disease-free survival; HR, hazard ratios; 95% CI, 95% confidence interval; NIH, National Institute of Health; TCGA-LIHC, The Cancer Genome Atlas Liver Hepatocellular Carcinoma. The top four genes belong to the HepatoPredict score and the bottom five belong to the 5-gene score. All genes were tested in the Danish cohort (Rigshospitalet), the TCGA-LIHC cohort, and the NIH cohort.

## DISCUSSION

None of the included genes were significantly linked to overall survival in the Danish cohort. *CLU* and *SPRY2* were significantly associated with overall survival in the TCGA-LIHC and NIH cohort, respectively. Four genes from the 5-gene score were linked to overall survival in the TCGA-LIHC cohort but not in the NIH cohort. No genes were found to be associated with disease-free survival in the three cohorts.

In previous separate studies of the individual genes, high expression of *HN1, RAN, KRT19,* and *CAPSN1* were associated with both worse overall survival and increased recurrence rates after liver resection or transplantation for HCC.^19^, ^20^, ^21^, ^22^, ^23^, ^24^, ^25^ Moreover, high expression of *DPT, RAMP3,* and *SPRY2* were associated with both better overall survival and recurrence-free survival.^26^, ^27^, ^28^, ^29^ High *TAF9B* expression was associated only with worse overall survival.^30^ Furthermore, high *CLU* expression was associated with worse overall survival in HCC patients treated with chemotherapy.^31^ However, other studies showed no significant prognostic value of *CLU* expression after liver resection.^32^^,^^33^

In our study, *CLU* was found to be significantly associated with better overall survival in the TCGA-LIHC cohort, and *SPRY2* was associated with worse overall survival in the NIH cohort, both being the opposite of the previous findings.^29^^,^^31^ Both findings, furthermore, oppose the HepatoPredict report, which also indicated that high *CLU* expression was associated with poorer overall survival, while high *SPRY2* expression was associated with better overall survival.^12^ Furthermore, high expressions of *HN1*, *RAN*, and *TAF9B* were all found associated with worse overall survival and high expression of *RAMP3* was found associated with better overall survival in the TCGA-LIHC cohort, all consistent with our expectations. The gene expression measurements utilized for the analysis of the TCGA-LIHC, the NIH, and the Danish cohort differ in their quantification methods. The Danish cohort used TPM, TCGA-LIHC used RSEM sequencing counts, and the NIH cohort used log-transformed values of signal intensity. These differences are reflected in the variation in hazard ratios. Furthermore, the abovementioned genes showed statistically significant associations with overall survival, although the effect sizes for these associations were negligible in most cases. These findings suggest that the practical impact on overall survival is minimal.

The outcomes of the two scores might not be reproducible in the Danish cohort due to the difference in patient characteristics. In the Danish cohort, most patients (75%) developed HCC in a non-cirrhotic liver, whereas the HepatoPredict study included only patients with cirrhosis^12^ and 34% of patients in the 5-gene score study had cirrhosis.^11^ Moreover, in the Danish cohort more than half of the patients had no prior known liver disease, only 20% of patients had HCV and none had HBV. Additionally, 16% of patients in the Danish cohort had alcoholic liver disease. In the 5-gene score study, 22% had HCV and 22% had HBV. Furthermore, alcohol consumption accounted for 39% of the etiologies of HCC.^11^ In the HepatoPredict study, 49% had HCV, 12% had HBV, and 74% had simultaneous alcohol consumption.^12^ The differences in etiologies may cause differences in genetic profiles and thus result in our findings being different than the HepatoPredict and the 5-gene score studies.^13^

The HepatoPredict study generated a signature by a two-step machine learning process that resulted in a decision tree based on the expression of four genes and clinical parameters (largest nodule size, total tumor volume, and number of nodules).^12^ The fundamental nature of machine learning methods precludes us from replicating their algorithm. The 5-gene score was developed by a feature selection process which selected genes that worked well when considered together to predict outcome. Their formula also includes a variable, which was not disclosed.^11^ Due to insufficient details about the gene score calculation method, we were unable to reproduce their exact approach. This lack of information prevents us from testing the gene scores as they were applied in the original studies.

The gene selection process in these studies itself is problematic as they lack transparency regarding the specific criteria used. Although it was stated that the gene selection was based on the gene’s association with prognosis, the exact criteria were not specified, thus an objective evaluation is impossible. In the 5-gene score study, 103 genes were tested on 44 HCC samples, which is, similar to our study, a limited sample size. Analyzing a large number of genes with a small sample size may lead to false positive results or overfitting, which can compromise the reliability and generalizability of the findings. In addition to the analyzed genes, the 5-gene score study included two genes (KRT19 and EPCAM) from the literature for the gene selection process.^11^ HepatoPredict identified their genes from a systematic literature review before analyzing the gene expression in their pilot set.^12^ The inclusion of genes from the literature introduces the possibility of bias stemming from the selective reporting of previous studies.

Our study was a retrospective, single-center study with a small sample size. Thus, the statistical power and the ability to detect significant associations may be reduced. However, this study design allowed for a long follow-up period and almost complete data without loss to follow-up. Moreover, we followed the STREGA guidelines for reporting to increase transparency of our study. Testing of the gene scores in TGCA and NIH cohorts enabled us to investigate the gene scores in larger cohorts. Both of these studies^15^^,^^16^ originally generated genome-scale molecular profiles, which were then placed in dedicated public repositories for future reanalysis by the clinical and scientific communities.^17^^,^^18^ The original studies looked at global changes in the transcriptome, whereas we used the transcriptome data to extract our individual genes of interest and test their individual associations with patient outcome. These publicly available datasets were exclusively used to reproduce findings from our local cohort, specifically that there was no association between the expression of these individual genes with patient outcomes. Molecular analysis and data accessibility from all TCGA studies have been diligently coordinated by the National Cancer Institute and National Human Genome Research Institute (USA), ensuring compliance with ethical and regulatory bodies.^15^ Molecular analysis in the NIH HCC cohort was approved by corresponding Institutional Review Boards as described in the original publication.^16^ The TCGA-LICH and NIH cohorts were exclusively used to either support or refute our in-house observations that the expression of specific genes was not individually associated with patient outcomes in patient populations. Our goal was to determine if these prognostic associations were reproducible in independent cohorts, so we also tested prognostic associations in HCC populations without adjusting for baseline characteristics. Our results clearly indicated that the individual expression of these nine genes was not associated with patient outcomes. Adjusting for baseline characteristics within survival analyses should only be performed if we intended to discover novel prognostic biomarkers independent of established prognostic baseline characteristics, which was not the goal of our study. The baseline characteristics of the patients from these two cohorts are thus not relevant for this analysis.

A limitation is that one of the investigated gene scores was based on liver-transplanted patients, and our patients were all treated with resection and thus might have different characteristics. In addition, unknown patient characteristics in the TCGA-LIHC and NIH cohort, including cirrhosis and underlying liver disease, make it harder to compare the groups based on characteristics. Due to the use of different methods of analyses among the three investigated cohorts, the hazard ratios of the individual genes could not be made comparable between the groups. The hazard ratios close to 1 with narrow confidence intervals were related to the unit in which the gene expression is measured, e.g. the change in hazard ratio with a single unit increment. This also explains the differences in hazard ratios between the NIH, TCGA, and our cohort due to the use of different units. However, the *P* values reflected the statistical correlation regardless of unit. The concept of a prognostic gene score as an addition to the current staging and prognostic guidelines such as BCLC could be clinically relevant to improve the selection of patients for liver resection to obtain optimal outcomes. However, the gene scores do not seem to be universally reproducible and are at this stage not sufficiently developed to apply them clinically. Future studies should include larger and more diverse cohorts with traceable clinical follow-up. De *novo* transcriptomic analyses based on whole RNA sequencing would be a useful tool to be able to take population-specific transcriptomic alteration into account.

In conclusion, the individual genes tested in these cohorts of resected patients, did not demonstrate prognostic differentiation as suggested by other studies on different patient groups. Confounding factors are differences in patient characteristics as well as secondary effects due to circumstances not recorded during the studies. These potential effects are amplified by a small sample size of the Danish cohort. However, some of the included genes showed prognostic value and the clinical implications of this need further investigation. Larger studies with population-specific *de novo* transcriptomic analyses are needed.

## FUNDING

JK received grants from the Research Foundation of Rigshospitalet not related to this work.

SDN received research grants from Novo Nordisk Foundation and Independent Research Fund not related to this work.

JBA received research funding from Incyte and consultancies for AstraZeneca, Flagship Pioneering, and QED Therapeutics not related to this work.

HCP reports grants from the Danish Cancer Society, Independent Research Fund, the Research Foundation of Rigshospitalet, and Svend Andersen Foundation not related to this work.

## CREDIT AUTHORSHIP CONTRIBUTION STATEMENT

SDS contributed to investigation, data Curation, writing–original draft, writing–review and editing, visualization.

JK contributed to conceptualization, writing–review and editing, supervision, project administration.

SBN contributed to data curation, writing–review and editing, supervision.

JM contributed to investigation, writing–review and editing.

COR contributed to methodology, formal analysis, writing–review and editing.

SDP contributed to writingreview and editing.

JBA contributed to methodology, writing–review and editing.

HCP contributed to conceptualization, methodology, writing–review and editing, supervision, project administration.

## Declaration of competing interest

All other authors declared no conflict of interest.
